# LATE: Nicht jede Demenz ist Alzheimer – Diskussion einer neuen Krankheitsentität am Fallbeispiel: Zum aktuellen Stand der „limbic-predominant age-related TDP-43 encephalopathy“ (LATE)

**DOI:** 10.1007/s00115-020-00922-z

**Published:** 2020-05-14

**Authors:** Doreen Görß, Ingo Kilimann, Martin Dyrba, Sascha Nitsch, Bernd Krause, Stefan Teipel

**Affiliations:** 1 Sektion Gerontopsychosomatik und demenzielle Erkrankungen, Universitätsmedizin Rostock, Gehlsheimer Str. 20, 18147 Rostock, Deutschland; 2 Deutsches Zentrum für Neurodegenerative Erkrankungen (DZNE), Standort Rostock/Greifswald, Rostock, Deutschland; 3 Klinik für Nuklearmedizin, Universitätsmedizin Rostock, Gertrudenstraße 1, Rostock, Deutschland

**Keywords:** Demenz, Alzheimer, TDP-43, Proteinopathie, SNAP, Dementia, Alzheimer’s disease, TDP-43, Proteinopathy, SNAP

## Abstract

Die limbisch prädominante altersassoziierte TDP-43(Transactivation response(TAR)-DNA-binding protein 43 kDa)-Enzephalopathie (LATE) wurde kürzlich als eigene neuropathologische Entität im Demenzspektrum charakterisiert. Neuropathologische Veränderungen im Sinne von LATE wurden zuvor bereits als Komorbidität der Alzheimer-Krankheit (AD) beschrieben und spätestens seit 2008 auch als ein von der AD unabhängiger autoptischer Befund. Die Konzeptualisierung von LATE rückt nun die pathogenetische Bedeutung von limbischem TDP-43 als alternative oder komorbide Ursache einer der klinischen AD ähnlichen amnestischen Demenz in das Bewusstsein. LATE könnte divergierende klinische und Biomarkerbefunde erklären, bei denen eine ausgeprägte mnestische Störung ohne Amyloid- und Tau-Veränderungen im Sinne einer AD-Pathologie nachweisbar ist. Ob LATE eine eigenständige neuropathologische Entität darstellt oder eine regionale Ausprägung innerhalb des Spektrums der bekannten TDP-43-assoziierten neurodegenerativen Erkrankungen ist aktuell Gegenstand kontroverser Diskussionen. Die weitere, gezielte Erforschung von TDP-43-Proteinopathien ist davon unabhängig ein vielversprechender Forschungsansatz, um Wissenslücken in der Alzheimer- und Demenzforschung zu schließen. So könnte ganz praktisch die Anreicherung eines amnestischen Phänotyps in klinischen Studien zu amyloidzentrierten Therapien durch das erhöhte Risiko limbischer TDP-43-Komorbidität den Nachweis der klinischen Wirksamkeit erschweren. Dieser Artikel stellt den aktuellen Stand der Diskussion zu LATE vor und illustriert das Konzept und daraus abgeleitete klinische Überlegungen an einem Fallbeispiel.

## Abstract (en)

Limbic-predominant age-related TDP-43 (Transactivation response(TAR)-DNA-binding protein 43 kDa) encephalopathy (LATE) has recently been characterized as a distinct neuropathological entity within the spectrum of dementia. Neuropathological alterations in the sense of LATE were already previously described as a comorbidity to Alzheimer’s disease (AD) and it has been diagnosed independently from AD pathology in autopsy studies since 2008. The framework of LATE would account for the pathogenetic impact of limbic TDP-43 proteinopathy as a driver of amnestic dementia, either together with comorbid typical AD changes or as a distinct feature. The LATE possibly explains divergent clinical observations and biomarker results in patients suffering from severe amnestic impairment without biomarker evidence of AD-related amyloid and tau alterations. Whether LATE represents a distinct neuropathological entity or is part of the spectrum of neurodegenerative diseases associated with TDP-43 is currently a matter of debate. Further studies on the role of TDP-43 in the development of amnestic dementia are urgently needed. Thus, the enrichment of an amnestic phenotype in amyloid-centered therapeutic drug studies bears the risk of higher rates of patients with TDP-43 comorbidity, which could hinder the proof of efficacy in such trials. This article presents the current state of the discussion on LATE and illustrates the concept and the clinical considerations with a case study.

Im April 2019 erschien in *BRAIN* (Fachzeitschrift verlegt von Oxford University Press) der Bericht einer Konsensusgruppe zum Thema „limbic-predominant age-related TDP-43 (Transactivation response(TAR)-DNA-binding protein 43 kDa) encephalopathy“, abgekürzt „LATE“, der einen ausführlichen Überblick über eine möglicherweise neue neuropathologische Entität im Demenzspektrum gibt [18]. Der vorliegende Artikel stellt den aktuellen Stand der Diskussion zu LATE vor und illustriert den Einfluss des Konzeptes auf differenzialdiagnostische Überlegungen bei klinischem Verdacht auf eine Alzheimer-Krankheit (AD) an einem Fallbeispiel.

## Hintergrund

Hyperphosphoryliertes und ubiquiniertes TDP-43 (TAR-DNA-bindendes Protein 43kDa) wurde 2006 als charakteristisches pathologisches Merkmal bei über der Hälfte der Fälle mit frontotemporaler Lobärdegeneration (FTLD) und sporadischer amyotropher Lateralsklerose (ALS) identifiziert [21]. Die TDP-43-Proteinopathie ist allerdings keineswegs auf diese Erkrankungsentitäten beschränkt. Auch bei neurodegenerativen Erkrankungen wie z. B. der Lewy-Körper-Krankheit und der Alzheimer-Krankheit (AD) war TDP-43 teilweise nachweisbar. Aktuell wird diskutiert, ob pathologisches TDP-43 eine Kopathologie bei primär nicht-TDP-43-assoziierten neurodegenerativen Erkrankungen ist oder eine eigene klinisch-pathologische Entität und somit Komorbidität darstellt [10]. Häufig ist die TDP-43-Proteinopathie mit einer hippokampalen Sklerose assoziiert [2, 19, 20, 22]. Im Konsensuspapier wurde vorgeschlagen, mit pathologischem TDP-43 assoziierte neuropathologische Veränderungen als LATE-NC („neuropathological change“) zu bezeichnen. LATE-NC kann auch bei kognitiv Gesunden auftreten, bei denen eine Enzephalopathie neuropathologisch retrospektiv nicht zu verifizieren ist [10]. Der neuropathologisch charakterisierten Entität „LATE-NC“ ist klinisch ein amnestischer Phänotyp „LATE“ zugeordnet [18]. Die Arbeit der Konsensusgruppe stärkt den Stellenwert von Biomarkern in der Demenzdiagnostik. LATE-NC wird vorerst jedoch eine neuropathologische Diagnose bleiben, da TDP-43-Biomarker bisher fehlen.

## LATE-NC: klinische und neuropsychologische Merkmale

Die limbisch prädominante, altersassoziierte TDP-43-Enzephalopathie (LATE) stellt möglicherweise eine neue Krankheitsentität unter dem Dach der neurodegenerativen Erkrankungen mit demenziellem Syndrom dar [18]. Patienten, bei welchen autopsiegesichert neuropathologische Veränderungen im Sinne von LATE-NC nachgewiesen wurden (mit als auch ohne Kopathologie), zeichneten sich durch folgende Charakteristika aus: Neuropsychologisch bestand vorwiegend ein amnestisches Syndrom, welches in ein demenzielles Syndrom mündete. Dabei war, wie bei AD, eine Störung des episodischen Gedächtnisses, häufig kombiniert mit Einschränkungen weiterer Domänen, festzustellen [17, 30]. Die als LATE-NC klassifizierten Patienten zeigten keine typischen Symptome einer FTLD oder primär progressiven Aphasie (PPA), denen histopathologisch häufig eine TDP-Pathologie zugrunde liegt.

Im Vergleich zu AD-Patienten scheinen LATE-NC-Patienten einen langsameren kognitiven Abbau zu erleiden. Demgegenüber zeigten Patienten mit AD-typischen neuropathologischen Veränderungen und komorbider TDP-43-Proteinopathie eine höhere Wahrscheinlichkeit der kognitiven Verschlechterung, als Personen mit AD-Pathologie ohne zusätzlichen TDP-43-Nachweis [11]. Außerdem gibt es Hinweise, dass Patienten mit einer Komorbidität aus TDP-43-Pathologie und AD eher Verhaltensauffälligkeiten wie Aggression oder Agitation zeigen als Patienten mit isolierter AD [27].

## LATE-NC: neuropathologische und bildgebende Befunde

Als neuropathologische Veränderungen im Sinne von LATE-NC wird der Nachweis von phosphoryliertem und fehllokalisiertem TDP-43 angesehen, welches seine normale Immunreaktivität verloren hat. LATE-NC-assoziierte TDP-43-Proteinopathien nehmen in Autopsiefunden mit dem Alter zu [12, 18, 29]. Der Altersgipfel der Prävalenz von LATE-NC scheint später zu sein als der anderer TDP-43-Pathologien. Ein Stadienschema definiert die Ausbreitung von TDP-43 von Amygdala über Hippokampus zum Gyrus frontalis medialis in drei Stadien [11, 17]. Die frühe Einführung eines solch stark vereinfachten Stadienschemas wurde zugleich vielfach kritisiert, ebenso die Abgrenzung von LATE-NC zur FTLD-TDP generell. LATE-NC wird von einigen Autoren vielmehr als Bestandteil des FTLD-TDP/ALS-Spektrums diskutiert. Ob die pathologischen TDP-43-Veränderungen bei FTLD-TDP biochemisch den TDP-Veränderungen bei anderen Erkrankungen, insbesondere bei komorbider TDP-43-Proteinopathie und AD, entsprechen, ist aktuell unklar [10]. Zwar ist der Nachweis einer hippokampalen Sklerose nicht spezifisch für LATE-NC, doch wurde diese in der Vergangenheit vielfach in Zusammenhang mit Non-AD-Demenz-Syndromen beschrieben [2]. In etwa der Hälfte der LATE-NC-Fälle war die hippokampale Sklerose nur einseitig nachweisbar, was sie von der FTLD mit TDP-43-Proteinopathie unterscheidet [18]. Das in vivo nachweisbare Korrelat der Sklerose stellt eine Atrophie in der strukturellen Bildgebung bzw. den verminderten Glukosemetabolismus im FDG-PET dar. Bei Patienten, deren Autopsiebefund einer LATE-NC entsprach, wurde ante mortem (mittels MRT) eine Atrophie des medialen Temporallappens analog zur Alzheimer-Krankheit festgestellt. Die hippokampale Atrophie scheint jedoch ausgeprägter zu sein, als bei AD. Post-mortem-MRT-Studien konnten zeigen, dass das zerebrale Atrophiemuster jeweils mit der Lokalisation der TDP-43-Proteinopathie übereinstimmte [18]. In longitudinalen MRT-Studien zeigte sich interessanterweise kein direkter Zusammenhang von Amyloid oder Tau mit einer Hippokampusatrophie bei Patienten mit komorbider LATE-NC und AD [5, 28].

## LATE-NC: genetische Risikofaktoren

Soweit heute bekannt, wurden fünf Gene identifiziert, welche mit pathologischen Veränderungen im Sinne von LATE-NC assoziiert sind: *ABCC9, APOE, GRN, KCNMB2, TMEM106B*. Bekanntermaßen sind *GRN*- und *TMEM106B*-Mutationen ebenfalls mit der FTLD-TDP-Proteinopathie assoziiert, was die Diskussion der Abgrenzung von LATE-NC zu bereits klassifizierten TDP-43-Proteinopathien kritisch antreibt. Ebenfalls zu bemerken ist, dass *APOE4* ein bekannter Risikofaktor für die Entwicklung einer AD als auch der vaskulären Demenz ist [18, 24].

## Die AT(N)-Klassifikation der AD

Im Jahr 2016 wurde die AT(N)-Klassifikation der AD vorgestellt [7, 8]. Obwohl sie für den wissenschaftlichen Kontext entwickelt wurde, kann sie auch bei klinischen Fragestellungen zu differenzialdiagnostischen Überlegungen herangezogen werden. Das AT(N)-System dichotomisiert auf individueller Basis sieben Biomarker innerhalb dreier Kategorien. Die Kategorie „A“ bezieht sich auf den Amyloidstatus (ermittelt über Liquor-Aβ42 oder Amyloid-PET), „T“ auf die Ergebnisse von Tau (Liquor-Phospho-Tau oder Tau-PET), „N“ steht für Neurodegeneration (festgestellt mittels MRT, Liquor-Gesamt-Tau oder FDG-PET). Jeder der Biomarker wird jeweils als positiv oder negativ evaluiert, sodass sich bspw. ein Status A^+^/T^−^/(N^+^) ergibt. Zusammengefasst werden alle Konstellationen mit A^+^ dem Alzheimer-Kontinuum zugeordnet. Als Normalzustand werden gänzlich unauffällige Biomarker (A^−^T^−^N^−^) angesehen. A^−^ mit mindestens einem weiteren positiv bewerteten Biomarker wird als pathologische Veränderung, welche nicht Alzheimer-typisch ist, eingestuft. Amyloid und Tau gelten als Alzheimer-definierend, während Neurodegeneration unspezifisch ist. Gemäß der Logik des AT(N)-Systems sollte die Gruppe der Patienten mit Demenz und Biomarkerstatus A^−^T^−^(N^+^) neben anderen Non-AD-Pathologien auch reine LATE-NC-Fälle beinhalten. Denkbar ist eine Erweiterung des Schemas, sofern neue Biomarker in vivo bestimmbar sind.

## Kasuistik

Ein Fallbeispiel soll die durch das Konzept „LATE-NC“ erweiterten differenzialdiagnostischen Überlegungen bei einer Patientin mit A^−^T^−^(N^+^)-Status illustrieren. Eine 55-jährige Patientin stellte sich 2015 erstmalig zur Abklärung einer Gedächtnisstörung vor. Sie berichtete von einer Orientierungs- und Aufmerksamkeitsstörung sowie einer Wortfindungsstörung. Klinisch-neurologisch ergaben sich keine pathologischen Befunde.

### Neuropsychologische Befunde

**Figure 1:**
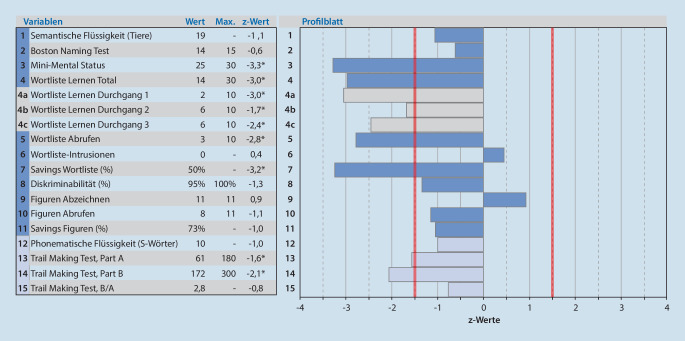

In der Testung 2019 erreichte die Patientin im Mini-Mental-Status-Test (MMST) 25 von 30 Punkte. Damit zeigte sich eine langsame Progredienz zu den Voruntersuchungen (MMST 2018: 26 Punkte, 2016: 29 Punkte). In der CERAD-Plus-Batterie [16, 25] war vorwiegend eine mnestische Störung festzustellen (Abb. 1, Variablen 4 bis 8). Darüber hinaus bestanden keine relevanten Defizite der Exekutivfunktionen oder der Visuokonstruktion (Abb. 1, Variablen 13, 14 und 9). Bei fehlender relevanter Einschränkung der Alltagskompetenz wurde syndromal 2015 eine leichte kognitive Störung („mild cognitive impairment“, MCI) gemäß der NIA-AA-Kriterien [1] diagnostiziert.

### Biomarker und Bildgebung

Ätiologisch bestand der Verdacht auf eine AD, sodass die weiterführende Diagnostik erfolgte (Tab. 1). Bei der Patientin ergab sich das Biomarkerprofil A^−^T^−^(N^+^). Folglich war eine pathologische Veränderung zu objektivieren, jedoch nicht spezifisch für eine Alzheimer-Krankheit.

**Table 1:** 

	Liquor		PET		cMRT	Rating
*A*	Aβ42:	2015: normwertig	Amyloid-PET:	Unauffällig	–	*A*^*−*^
		2018: normwertig				
*T*	Phospho-Tau:	2015: normwertig	Tau-PET:	Nicht durchgeführt	–	*T* ^*−*^
		2018: normwertig				
*(N)*	Gesamt-Tau:	2015: normwertig	FDG-PET:	Hypometabolismus temporal, parietal^a^	2015: Hippokampusatrophie	*(N)*^*+*^
		2018: normwertig			2019: Hippokampusatrophie^a^	

In der Lumbalpunktion ergaben sich keine entzündlichen Veränderungen; alle Destruktionsparameter waren normwertig

**Figure 2:**
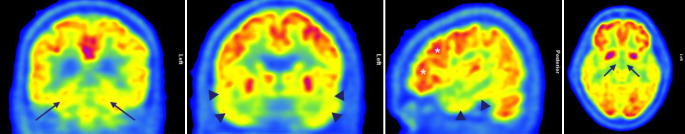

**Figure 3:**
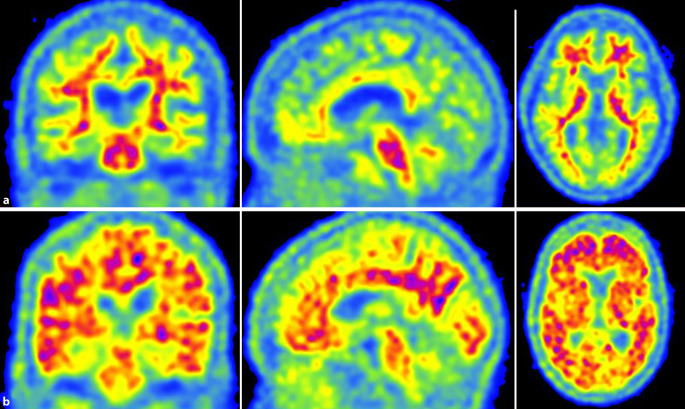

^a^Befunde der PET/CT und cMRT siehe Abb. 2, 3, 4 und 5

**Figure 4:**
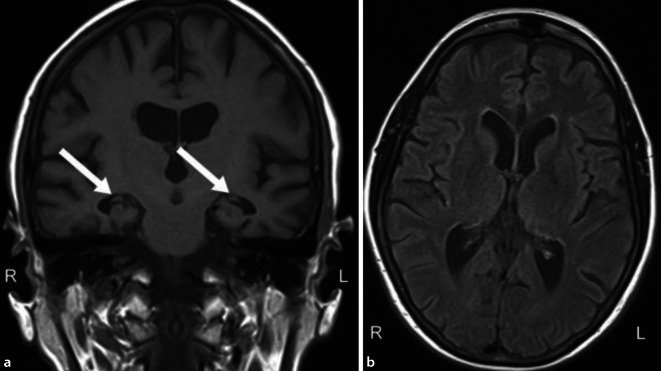

**Figure 5:**
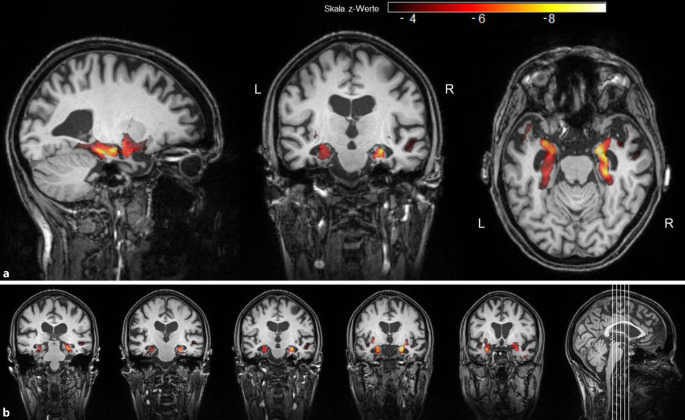

Bei der Patientin wurde zweimalig Liquor punktiert. Die Destruktionsparameter waren normwertig. In der Fluordesoxyglukose-Positronenemissionstomographie (FDG-PET) 2015 zeigte sich ein reduzierter Glukosemetabolismus temporal und parietal beidseits, linksbetont (Abb. 2). Die Flutemetamol-PET 2019 ergab keinen Nachweis von β‑Amyloid (Abb. 3). In der strukturellen Bildgebung kam eine Hippokampusatrophie beidseits (MTA3°, „medial temporal lobe atrophy score“) zur Darstellung (Abb. 4). Das Hippokampusvolumen der Patientin war im Vergleich zu einer altersgematchten Kontrollgruppe (*n* = 20 gesunde, weibliche Kontrollen) deutlich reduziert. Abb. 5 zeigt das Ausmaß und die Lokalisierung der Atrophie.

### Differenzialdiagnostische Überlegungen

Der amnestische Phänotyp unserer Patientin wäre typisch für eine AD. Auch die nachgewiesene Neurodegeneration wäre hiermit gut vereinbar. Gegen die Diagnose einer AD und für eine Non-AD sprechen die unauffälligen Amyloid- und Tau-Werte [9]. Der negative Liquorstatus wäre gut im Rahmen einer FTLD erklärbar. Klinisch imponiert die behaviorale Variante der FTLD (bvFTLD) durch neuropsychologische Symptome. Neben Verhaltensauffälligkeiten wie Impulsivität oder Enthemmung können Apathie, sprachliche Stereotypien oder veränderte Ernährungsgewohnheiten auftreten [23]. Anamnestisch ergibt sich bei unserer Patientin hierfür jedoch kein Anhalt. Gegen eine bvFTLD sprechen außerdem der amnestische Phänotyp (bei bvFTLD stärker Exekutivfunktionen beeinträchtigt), der langsame Verlauf, der fehlende Nachweis eines frontalen Hypometabolismus in der FDG-PET und der fehlende Nachweis einer fronto-/temporalen Atrophie in der strukturellen Bildgebung [15]. Eine Sprachvariante einer FTLD ist durch die diesbezüglich unauffällige kognitive Testung ausgeschlossen.

Differenzialdiagnostisch ist weiterhin eine PART („primary age-related tauopathy“) zu diskutieren, eine leichte mnestischen Störung in Verbindung mit einer Temporallappenatrophie und Tauopathie (neuropathologisch nachgewiesen), ohne Nachweis von β‑Amyloid [3, 14]. Typisch hierfür ist die Beschreibung einer kognitiven Störung durch die Patienten selbst, bei keinen oder nur milden nachweisbaren kognitiven Einbußen [13]. PART ist eine neuropathologische Diagnose. Diagnosekriterien ante mortem existieren nicht. Die Gedächtnisstörung und die Abwesenheit einer Amyloidpathologie in Verbindung mit der Atrophie bei unserer Patientin wären mit dem Vorliegen einer PART vereinbar. Allerdings sprechen die unauffälligen Phospho-Tau-Werte im Liquor gegen das Vorliegen einer Tauopathie. Zumindest konnte bei Patienten mit FTLD gezeigt werden, dass Tau-Werte im Liquor ante mortem mit einer post mortem festgestellten Tauopathie korrelierten [6]. Zusammenfassend diagnostizierten wir eine leichte kognitive Störung (MCI) unklarer Genese. Außerhalb klinischer Studien besteht keine medikamentöse Therapieoption für MCI. Sollte die kognitive Störung weiter zunehmen und in ein demenzielles Syndrom münden, sehen wir bei fehlendem Hinweis auf eine AD eine medikamentöse Therapie mit Acetylcholinesterase-Inhibitoren für nicht gerechtfertigt an. Die ausführliche ärztliche und sozialmedizinische Beratung solcher Patienten ist obligat. Wir haben die Patientin zusätzlich in eine Beobachtungsstudie eingeschlossen.

Für eine limbisch prädominante TDP-43-Proteinopathie sprechen die klinische Präsentation (mnestische Störung, langsame Progredienz), die Befunde der FDG-PET und der MRT mit Hinweisen für eine Neurodegeneration mit Fokus im medialen Temporallappen als auch der fehlende Amyloidnachweis in Liquor und PET, sowie der fehlende Tau-Nachweis im Liquor. Untypisch für LATE-NC ist das Lebensalter der Patientin. Diese war bei Erstvorstellung 55 Jahre alt und damit deutlich jünger als die meisten beschriebenen LATE-NC-Fälle. Es gibt zwar bisher keine festgelegten Altersgrenzen, jedoch scheint LATE eine Erkrankung eher des hohen Lebensalters zu sein (über 80 Jahre). Die Prävalenz bei jüngeren Erwachsenen könnte jedoch höher sein, als bisher angenommen. Es herrscht Einigkeit über die die Notwendigkeit der Einbeziehung von Daten jüngerer Patienten in entsprechende Analysen [26].

## Schlussfolgerungen

Die exakten pathophysiologischen Mechanismen, welche den aktuell bekannten neurodegenerativen Erkrankungen zugrunde liegen, sind bisher nicht hinreichend aufgeklärt. Es ist anzunehmen, dass verschiedene Pathologien häufig koexistieren, insbesondere im hohen Lebensalter [4, 18]. Auch wenn gegenwärtig unklar ist, ob LATE tatsächlich eine distinkte Erkrankung darstellt, wird den aktuellen Erkenntnissen aus der Grundlagenforschung mit der Konzeptualisierung von LATE-NC und LATE Rechnung getragen. Wesentliche Fortschritte im Verständnis dieser Proteinopathie werden jedoch erst durch die Verfügbarkeit von Biomarkern für TDP-43 möglich sein. Die aktuelle Diskussion unterstreicht die Notwendigkeit prospektiver Autopsiestudien, wie sie z. B. durch das Deutsche Zentrum für Neurodegenerative Erkrankungen (DZNE) im Rahmen des Brain-Bank-Projektes bundesweit durchgeführt werden.

## Zusammenfassung und Ausblick

LATE-NC kann eine mögliche Erklärung für die Divergenz von klinischen Befunden und pathologischen Befunden (ausgeprägte mnestische Störung, aber kaum/kein Nachweis von Amyloid und Tau) bei klinisch diagnostizierten AD-Patienten sein. Für die Alzheimer-Forschung, gerade mit der möglichen Perspektive zukünftiger antiamyloidzentrierter Therapien, wird die Charakterisierung komorbider neurodegenerativer Veränderungen eine große Rolle spielen. Sollte eine Stratifizierung von Patienten hinsichtlich einer TDP-43-Proteinopathie gelingen, wäre dies für zukünftige klinische Studien von enormer Bedeutung. Zunächst könnte die Analyse von Subgruppen für bestehende Therapieansätze genutzt werden, um den Effekt der eingesetzten Präparate präziser für Subgruppen zu bestimmen. Zum anderen könnte TDP-43, oder damit assoziierte Krankheitsmechanismen, ein mögliches Target für zukünftige Therapieansätze darstellen. Auch klinisch ist LATE-NC relevant, da die neuropathologischen Veränderungen bei Menschen mit nachgewiesener Alzheimer-Pathologie eine häufige Komorbidität darstellen und die Wahrscheinlichkeit des Auftretens einer Demenz erhöhen. Die Erkenntnisse bezüglich der demenzassoziierten Proteinopathien (einschließlich LATE-NC) stammen zu großen Teilen aus Autopsiestudien. Erfahrungsgemäß ist dies in der Allgemeinbevölkerung ein unbehagliches Thema. Die qualifizierte Information über den Nutzen derartiger Studien für die zukünftige Entwicklung wirksamer Therapien kann helfen, Studienteilnehmer zu rekrutieren. Daher hat das DZNE mit universitären Partnern eine Brain Bank aufgebaut. Medizinisches Fachpersonal und insbesondere Ärzte können einen Beitrag für die Forschung leisten, indem sie Patienten über die Möglichkeit der Teilnahme an derartigen Studien informieren.

## Fazit für die Praxis

Im klinischen Alltag haben für neurodegenerative Demenzerkrankungen unverändert die Diagnosesysteme ICD-10 und DSM‑5 Gültigkeit. Die Bedeutung der Biomarker im diagnostischen Prozess wird mit Diskussion um LATE(limbic-predominant age-related TDP-43 [Transactivation response(TAR)-DNA-binding protein 43 kDa] encephalopathy)-NC(„neuropathological change“) unterstrichen. Die neusten Erkenntnisse sollten Motivation sein, allen Patienten weiterhin eine differenzierte Diagnostik zukommen zu lassen, die zur Beratung und Prognose und zu differenzierten therapeutischen Entscheidungen beiträgt. Außerdem sollten wir unsere Patienten über die Möglichkeiten einer Studienteilnahme informieren. Das Spektrum reicht von Beobachtungsstudien über pharmakologische und nichtpharmakologische Interventionsstudien bis hin zu Autopsiestudien.
